# NF-κB p65 and p105 implicate in interleukin 1β-mediated COX-2 expression in melanoma cells

**DOI:** 10.1371/journal.pone.0208955

**Published:** 2018-12-18

**Authors:** Nanako Kitanaka, Rei Nakano, Taku Kitanaka, Shinichi Namba, Tadayoshi Konno, Tomohiro Nakayama, Hiroshi Sugiya

**Affiliations:** 1 Laboratories of Veterinary Biochemistry, Nihon University College of Bioresource Sciences, Kameino, Fujisawa, Kanagawa, Japan; 2 Laboratory for Cellular Function Conversion Technology, RIKEN Center for Integrative Medical Sciences, Suehiro-cho, Tsurumi, Yokohama, Kanagawa, Japan; 3 Veterinary Radiotherapy, Nihon University College of Bioresource Sciences, Kameino, Fujisawa, Kanagawa, Japan; Chang Gung University, TAIWAN

## Abstract

Inflammatory and microenvironmental factors produced by cancer cells are thought to directly or indirectly promote cancer cell growth. Prostaglandins, including prostaglandin E2, have key roles as a microenvironment factor in influencing the development of tumors, and are produced by the rate limiting enzyme cyclooxygenase 2 (COX-2). In this study, we used canine melanoma cells treated with the proinflammatory cytokine interleukin 1β (IL-1β) and investigated the transcriptional factor nuclear factor-κB (NF-κB) signaling in IL-1β-induced COX-2 expression. IL-1β induced prostaglandin E2 release and COX-2 mRNA expression in a time- and dose-dependent manner. In the cells treated with the NF-κB inhibitors BAY11-7082 and TPC-1, IL-1β-mediated prostaglandin E2 release and COX-2 mRNA expression were inhibited. IL-1β also provoked phosphorylation of p65/RelA and p105/NF-κB1, which are members of the NF-κB families. The IL-1β-induced phosphorylation of p65 and p105 was attenuated in the presence of both NF-κB inhibitors. In melanoma cells transfected with siRNA of p65 or p105, IL-1β-mediated COX-2 mRNA expression was inhibited. These findings suggest that canonical activation of NF-κB signaling plays a crucial role for inflammatory states in melanoma cells.

## Introduction

Inflammation is associated with the promotion of cancer development [1–4]. Inflammatory and microenvironmental factors, produced by the cancer cell themselves, the stroma, or tumor-infiltrating leukocytes, have been considered to directly or indirectly promote cancer cell growth. Prostaglandins are implicated in carcinogenesis by enhancing cancer cell survival, proliferation, invasion, and angiogenesis [5, 6].

Prostaglandins are produced from arachidonic acid. Cyclooxygenases (COXs) are catalysing enzymes for the conversion, which exist in two forms, COX-1 and COX-2 [7]. COX-1 is constitutively expressed in most tissues, whereas COX-2 is inducible in response to several stimuli, such as cytokines, growth factors, and tumor promoters [8–10]. COX-2 overexpression has been reported in several cancers in humans [10, 11]. The inhibition of COXs by COX inhibitors including nonsteroidal anti-inflammatory drugs (NSAIDs) has been demonstrated to reduce the incidence and metastasis of various solid tumors and mortality [12–14]. These observations imply that the activation of COX-2 and subsequently produced prostaglandins are associated with the enhancement of cancer cell survival, growth, migration, angiogenesis, and immunosuppression [5].

The effects of COX-2 in melanomas are largely thought to be caused by its role in the production of prostaglandins, especially prostaglandin E_2_ [5]. In melanoma cells, prostaglandin E_2_ has been demonstrated to promote cell migration, because prostaglandin E_2_ receptor agonists stimulated cell migration while a prostaglandin E_2_ receptor antagonist suppressed its migratory capacity [15]. Furthermore, in the melanoma cells overexpressing COX-2, an increased in prostaglandin E_2_ levels and expression of prostaglandin E_2_ receptors resulted in the promotion of cell migration [16]. These observations suggest that prostaglandin E_2_ produced via COX-2 expression in melanoma cells functions as an autocrine or paracrine factor. Within the tumor microenvironment, prostaglandin E_2_ produced by cancer cells has been demonstrated to induce immunosuppression through the inhibition of differentiation, infiltration and activation of dendritic cells, induction of monocytes into an M2 macrophage phenotype, and induction of myeloid-derived suppressor cell differentiation [6].

The transcription factor nuclear factor-κB (NF-κB) regulates inflammatory responses by enhancing the expression of specific cellular genes, which further links to the promotion of carcinogenesis [17, 18]. COX-2 is a major molecular target of NF-κB. Various inflammatory stimuli and mediators have been demonstrated to increase COX-2 expression via the activation of NF-κB, thus eliciting inflammation and consequent tumorigenesis [19–23]. In mammals, the NF-κB family consists of five members: RelA (p65), RelB, Rel (cRel), NF-κB1 (p50 and its precursor p105), and NF-κB2 (p52 and its precursor p100) [24, 25]. The five family members associate with each other to form homodimers or heterodimers with distinct functions [26]. NF-κB signaling is composed of two distinct pathways: canonical and non-canonical pathways [27]. The canonical pathway mediates inflammatory responses, and the non-canonical pathway contributes to immune cell differentiation and maturation as well as secondary lymphoid organogenesis [27].

Oral canine malignant melanoma is a spontaneously occurring aggressive tumor [28]. The canine melanoma is highly metastatic and usually associated with a poor prognosis, and has a propensity to behave in a biologically aggressive manner similar to human melanoma. Therefore, the canine melanoma is considered a suitable model for human melanoma [29, 30]. Human and canine melanomas also share multiple molecular similarities and signaling pathways including the regulation of COX-2 expression, and upregulation of COX-2 expression in melanoma cells has been demonstrated [31, 32]. In this study, we demonstrated that NF-κB p65 and p105 were implicated in IL-1β-mediated COX-2 expression in canine melanoma cells.

## Materials and methods

### Materials

Canine melanoma cells (MCM-N1 cell line; 13-years-old male dog; chromosome number, 2n = 74) were purchased from DS Pharma Biomedical Co., Ltd. (Osaka, Japan). Lipofectamine 2000 and TRIzol were purchased from Life Technologies Co. (Carlsbad, CA). PrimeScript RT Master Mix, SYBR Premix Ex Taq II, Thermal Cycler Dice Real Time System II, and TP900 DiceRealTime v4.02B were purchased from TaKaRa Bio Inc. (Shiga, Japan). Rabbit polyclonal anti-COX-1 and anti-COX-2 antibodies were obtained from Abcam (Cambridge, UK). Mouse monoclonal anti-human lamin A/C (4C11) antibodies and Rabbit monoclonal anti-human phosphorylated p65 (93H1), anti-human total p65, anti-human phosphorylated p105, and anti-human total p105 antibodies were purchased from Cell Signaling Technology Japan, K.K. (Tokyo, Japan). Mouse monoclonal anti-mouse β-actin antibody (AC74) was obtained from Sigma-Aldrich Inc. (St Louis, MO). Horseradish peroxidase-conjugated (HRP-conjugated) anti-rabbit IgG and anti-mouse IgG antibodies, ECL Western Blotting Analysis System and ImageQuant LAS 4000 mini were purchased from GE Healthcare (Piscataway, NJ). Mini-PROTEAN TGX gel and polyvinylidene difluoride (PVDF) membranes were obtained from Bio-Rad (Hercules, CA). Block Ace and complete mini EDTA-free protease inhibitor mixture were purchased from Roche (Mannheim, Germany). An enzyme-linked immunosorbent assay (ELISA) kit was purchased from Cayman Chemical Co. (Ann Arbor, MI). The p65, p105, and scramble siRNAs were purchased from Sigma-Aldrich Inc. (St Louis, MO). Dulbecco's modified Eagle’s medium with 1 g/L glucose (DMEM-LG), phenylmethanesulfonyl fluoride (PMSF), sodium fluoride, and 4-(2-hydroxyethyl)-1-piperazineethanesulfonic acid (HEPES) were purchased from Wako Pure Chemical Industries, Ltd. (Osaka, Japan). StatMate IV was purchased from ATMS (Tokyo, Japan).

### Cell culture

Canine melanoma cells were maintained in static culture in DMEM-LG supplemented with 10% fetal bovine serum (FBS), in an incubator with 5% CO_2_ and at 37°C. The medium was changed once a week. When the cells reached 90–95% confluency, the cells were harvested with 0.25% trypsin-EDTA and suspended with CELLBANKER 1 plus medium at a density of 2 × 10^6^ cells/500 μL for cryopreservation. The cell suspension (500 μL) was placed into sterilized serum tube, which were placed in a freezing vessel (BICELL) and cryopreserved at −80°C. Before experiments, the tubes were removed from the BICELL vessel and immersed in a water bath at 37°C. The thawed cell suspension was transferred into a centrifuge tube containing DMEM-LG with 10% FBS and centrifuged at 300 ×*g* for 3 min. The pellet was resuspended in DMEM-LG with 10% FBS and transferred into a 75-cm^2^ culture flask. Static culture was then carried out under the same conditions as prior to cryopreservation. Cells were harvested using 0.25% trypsin-EDTA once they reached approximately 90% confluency, and the collected cells were seeded at a density of 1 × 10^6^/75-cm^2^ culture flask.

### Real-time RT-PCR

Total RNA was extracted from canine melanoma cells with TRIzol reagent. The first-strand cDNA synthesis was performed with 500 ng of total RNA using PrimeScript RT Master Mix. Real-time RT-PCR was performed with 2 μL of the first-strand cDNA in 25 μL (total reaction volume) with SYBR Premix Ex Taq II and primers specific for canine COX-1 and -2, and the TATA box binding protein (TBP), a house keeping protein used as a control. Table 1 shows sequences of primers used for real-time RT-PCR. Real-time RT-PCR of no-template controls was performed with 2 μL of RNase- and DNA-free water. In addition, real-time PCR of a no-reverse transcription control was performed with 2 μL of each RNA sample. PCR was conducted using the Thermal Cycler Dice Real Time System II with the following protocol: 1 cycle of denaturing at 95°C for 30 s, 40 cycles of denaturing at 95°C for 5 s, and annealing/extension at 60°C for 30 s. The results were analyzed by the second derivative maximum method and the comparative cycle threshold (ΔΔCt) method using real-time RT-PCR analysis software. The amplification of TBP from the same amount of cDNA was used as an endogenous control, while cDNA amplification from canine melanoma cells at time 0 was used as a calibration standard.

**Table 1:** Primers used for Real-time RT-PCR.

Gene Name	Gene bank ID	Primer sequences
*COX-2*	NM_001003354.1	F: 5ʹ-TGTGTCTCATTAACCTGCATGTACC-3ʹ
		F: 5ʹ-CAGTGATATTTGCACCTGTGTCCTC-3ʹ
*COX-1*	NM_001003023.2	F: 5ʹ-ACGTGGCTGTGGAAACCATC-3ʹ
		R: 5ʹ-GGCATCAATGTCTCCATACAGCTC-3ʹ
*TBP*	XM_863452	F: 5'-ACTGTTGGTGGGTCAGCACAAG-3'
		R: 5'-ATGGTGTGTACGGGAGCCAAG-3'

### Western blotting

The melanoma cells were lysed with a lysis buffer containing 20 mM HEPES, 1 mM PMSF, 10 mM sodium fluoride, and a complete mini EDTA-free protease inhibitor cocktail at pH 7.4. Protein concentrations were adjusted using the Bradford method [33]. Extracted proteins were boiled at 95°C for 5 min in SDS buffer. Samples were loaded into separate lanes of 7.5% or 12% Mini-PROTEAN TGX gel and electrophoretically separated. Separated proteins were transferred to PVDF membranes, treated with Block Ace for 50 min at room temperature, and incubated with primary antibodies (COX-2 [1:1000], COX-1 [1:1000], p-p65 [1:1000], t-p65 [1:1000], p-p105 [1:1000], t-p105 [1:1000], and β-actin [1:10,000]) for 120 min at room temperature. After washing, the membranes were incubated with an HRP-conjugated anti-rabbit or anti-mouse IgG antibody (1:10000) for 90 min at room temperature. Immunoreactivity was detected using ECL Western Blotting Analysis System. Chemiluminescent signals of the membranes were measured using ImageQuant LAS 4000 mini.

### Immunocytochemistry

The protein localization was investigated by immunocytochemical analysis as reported previously [34]. The cells were seeded at a density of 3 × 105 cells/mL culture medium into a 35-mm glass bottom dish (Iwaki, Tokyo, Japan) treated with IL-1β. The cells were fixed with 4% paraformaldehyde (Nacalai Tesque Inc., Kyoto, Japan) for 15 min and processed for immunocytochemistry to examine the intra-cellular localization of t-p65 and lamin A/C. The fixed cells were permeabilized by incubation with 0.2% Triton X-100 (Sigma-Aldrich Inc.) for 15 min at room temperature. Non-specific antibody reactions were blocked for 30 min with Block Ace (DS Pharma Biomedical, Osaka, Japan). The cells were then incubated for 90 min at room temperature with anti-t-p65 rabbit antibody [1:500] and anti-lamin A/C mouse antibody [1:1000]. After the cells were washed with PBS containing 0.2% polyoxyethylene (20) sorbitan monolaurate, they were incubated and visualized with Alexa Fluor 488-conjugated F(ab′)2 fragments of goat anti-rabbit IgG (H+L) [1:1,000] and Alexa Fluor 594-conjugated F(ab′)2 fragments of goat anti-mouse IgG (H+L) [1:1,000] for 60 min in the dark at 25°C. The cells were also incubated with only secondary antibodies as a control for nonspecific binding of the antibodies. These samples were washed thrice with PBS containing 0.2% polyoxyethylene (20) sorbitan monolaurate, dried, mounted with ProLong Gold Antifade Reagent, and visualized using a confocal laser scanning microscope (LSM-510; Carl Zeiss AG, Oberkochen, Germany).

### Prostaglandin E_2_ assay

The cells were seeded at a density of 3.0 × 10^5^ cells per well in 6-well culture plates. The cells were treated with IL-1β, and subsequently culture supernatants were collected. Prostaglandin E_2_ concentrations in the culture supernatant were measured using an ELISA kit according to the manufacturer’s instructions.

### Transfection of siRNA

The siRNA transfection was performed as previously described, with slight modifications [34, 35]. Canine melanoma cells, seeded at a density of 1 × 10^5^ cells/35-mm dish or 5 × 10^5^ cells/90-mm dish, were transfected using Opti-MEM containing 5 μL/mL Lipofectamine 2000 and 100 nM p65, p105, or scramble siRNA for 6 h. After the transfection, the medium was changed to DMEM-LG containing 10% FBS, and the cultures were maintained in an incubator with 5% CO_2_ at 37°C for five days. The siRNA sequences are shown in Table 2. The efficiency of the siRNAs was determined by western blotting.

**Table 2:** Sequences for siRNA transfection.

Gene Name	Gene bank ID	siRNA sequences
*p65*	XM_014121307.2	GCAUCUCCCUGGUCACCAA
*p105*	AB183419.1	CUGCAAAGGUUAUUGUUCA

### Statistical analysis

The data from these experiments were presented as the mean ± standard error of measurement. Statistical analysis was performed using StatMate IV. The data from the time course study were analyzed using two-way analysis of variance, and the data from other experiments were analyzed using one-way analysis of variance.

## Results

### IL-1β mediates prostaglandin E_2_ release and COX-2 expression

**Fig 1:**
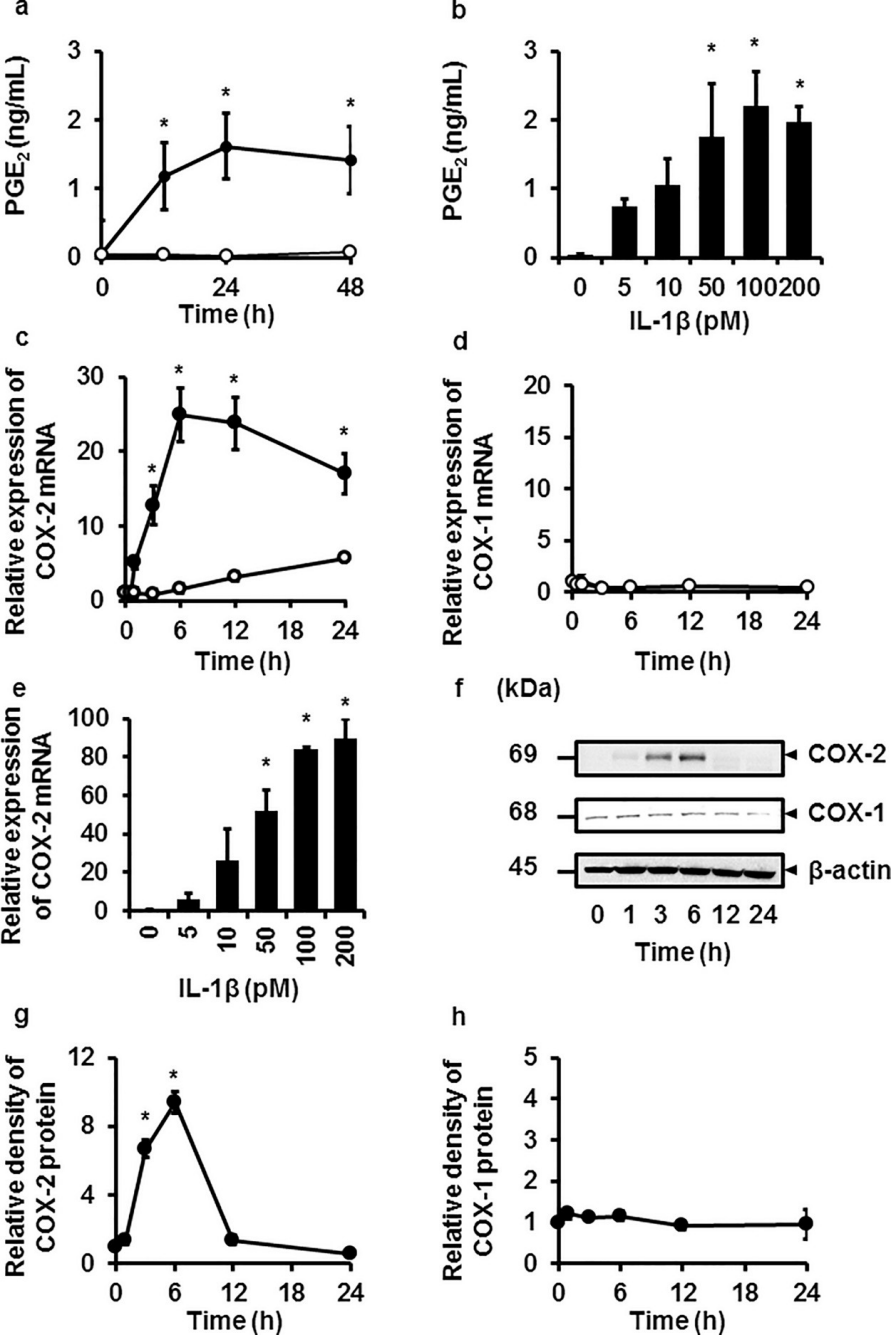
Prostaglandin E_2_ release, and COX-2 mRNA and protein expressions induced by IL-1β in canine melanoma cells. (**a, b**) After the treatment with (closed circle) or without (open circle) 100 pM IL-1β for the indicated time periods (**a**), or with the indicated concentrations of IL-1β for 48 h (**b**), prostaglandin E_2_ (PGE_2_) release was increased in a time- and dose-dependent manner. (**c, d, e**) In the cells treated with (closed circle) or without (open circle) 100 pM IL-1β for the indicated time periods (**c**), or with the indicated concentrations of IL-1β for 6 h (**d**), mRNA expression of COX-2 increased in a time- and dose-dependent manner, whereas IL-1β had no effect on COX-1 mRNA expression (**e**). (**f, g, h**) In the cells treated with IL-1β (100 pM), protein expressions of COX-1, COX-2, and β-actin (an internal standard) were examined (**f**). A time-dependent increase in the relative density of COX-2 expression was observed (**g**) but not that of COX-1 (**h**). Values are expressed as the mean ± S.E. of three independent experiments. *P < 0.05.

We first examined the effect of the proinflammatory cytokine IL-1β on prostaglandin E_2_ release in canine melanoma cells. Prostaglandin E_2_ release was provoked in the cells stimulated with IL-1β (100 pM) from 0 to 48 h in a time-dependent manner as shown in Fig 1A. In the cells stimulated with 0–200 pM IL-1β for 48 h, prostaglandin E_2_ release was provoked in a dose-dependent manner as shown in Fig 1B. We also checked the effect of IL-1β (100 pM) on the cell viability of canine melanoma cells. As shown in S1 Fig, IL-1β had no effect on the cell viability for 0 to 48 h. Since the production of prostaglandins is regulated by COX-1 and COX-2, which are rate-limiting enzymes, we examined the effect of IL-1β on the expressions of COX-1 and COX-2 mRNAs. IL-1β (100 pM) enhanced COX-2 mRNA expression in a time-dependent manner; the level peaked at 6 h (Fig 1C). On the other hand, IL-1β had no effect of COX-1 mRNA expression (Fig 1D). When cells were stimulated with various doses of IL-1β for 6 h, a dose-dependent enhancement of COX-2 mRNA was observed (Fig 1E). The dose range of IL-1β was similar to that for prostaglandin E_2_ release. Next, the effect of IL-1β on COX-2 protein expression was examined. IL-1β (100 pM) stimulated COX-2 protein expression in a time-dependent manner; the levels peaked at 6 h (Fig 1F and 1G). On the other hand, no change in COX-1 protein expression was observed in the cells stimulated with IL-1β (Fig 1F and 1H). These observations strongly suggest that IL-1β provoked prostaglandin E_2_ release via COX-2 expression in canine melanoma cells.

### NF-κB inhibitors attenuated IL-1β-mediated prostaglandin E_2_ release and COX-2 mRNA expression

**Fig 2:**
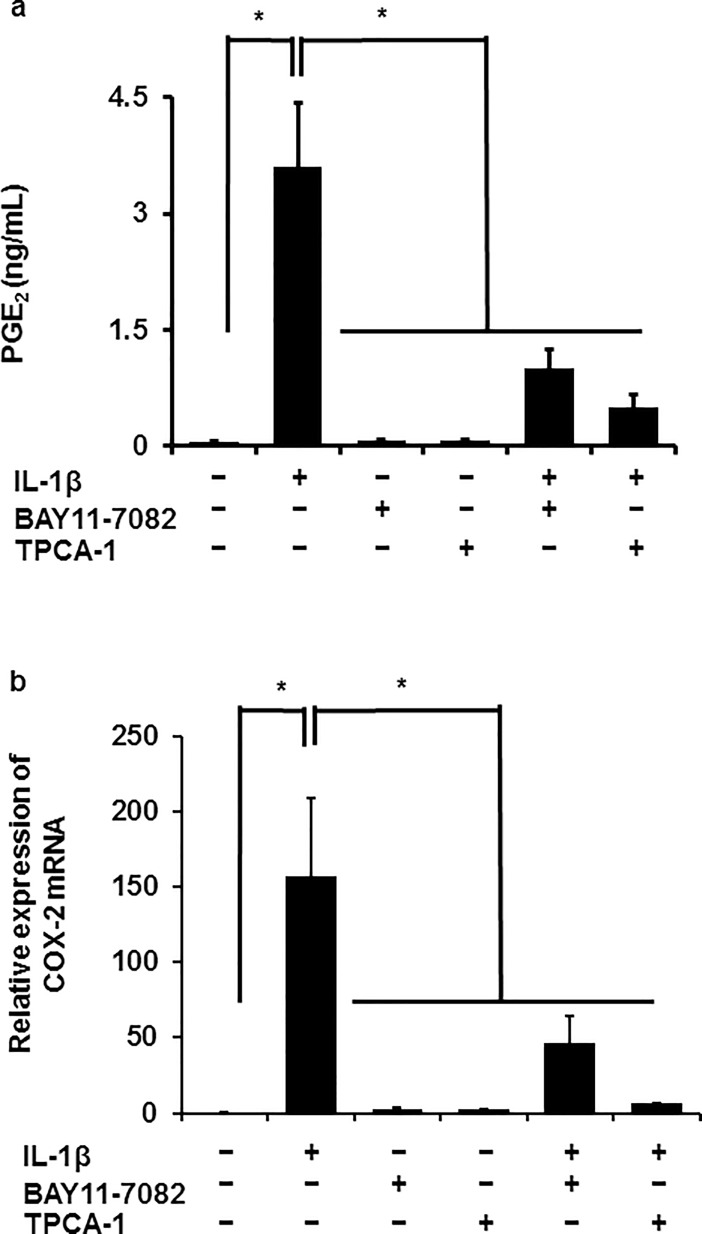
Inhibitory effects of NF-κB inhibitors on IL-1β-induced prostaglandin E_2_ release and COX-2 mRNA expression in canine melanoma cells. After pretreatment in the presence or absence of NF-κB inhibitors BAY11-7082 (10 μM) and TPCA-1 (10 μM) for 1 h, canine melanoma cells were further treated with or without 100 pM IL-1β for 48 or 6 h for the examination of prostaglandin E_2_ (PGE_2_) release (**a**) or COX-2 mRNA expression (**b**), respectively. Values are expressed as the means ± S.E. of three independent experiments. *P < 0.05.

Next, we examined the involvement of NF-κB signaling in IL-1β-mediated prostaglandin E_2_ release and COX-2 mRNA expression in canine melanoma cells by using NF-κB inhibitors. When the cells were pretreated with NF-κB inhibitors BAY11-7082 (10 μM) or TPCA-1 (10 μM) for 1 h and subsequently stimulated with IL-1β (100 pM) for 48 h, IL-1β-mediated prostaglandin E_2_ release was clearly reduced (Fig 2A). In the cells pretreated with the NF-κB inhibitors, IL-1β-mediated COX-2 mRNA was significantly attenuated (Fig 2B). These results suggest that NF-κB signaling is involved in IL-1β-mediated prostaglandin E_2_ release via COX-2 expression.

### IL-1β stimulates phosphorylation of p65 and p105

**Fig 3:**
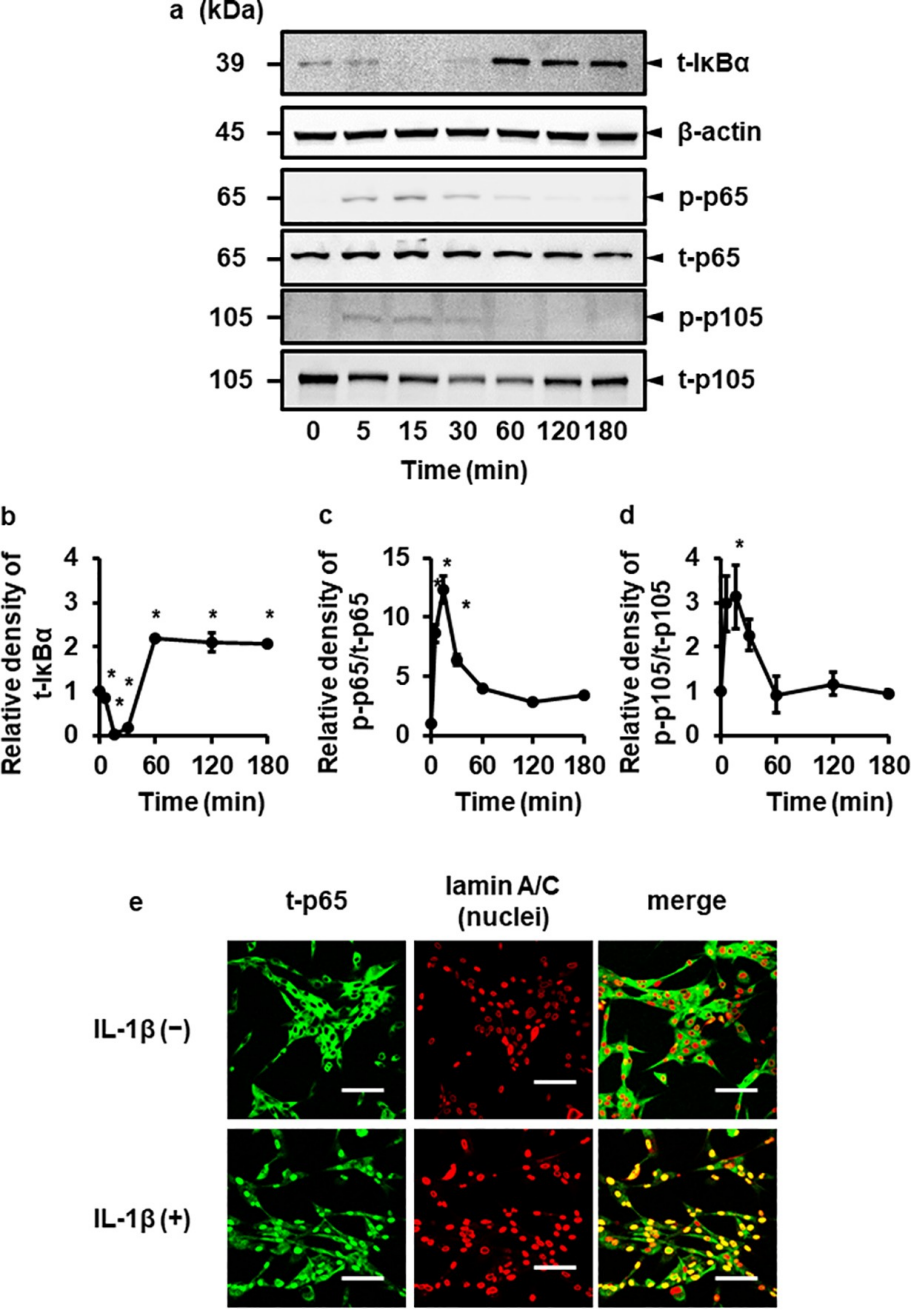
IL-1β induces the activation of canonical NF-κB pathway. Canine melanoma cells were exposed to 100 pM IL-1β for the indicated time periods. At the end of the incubation, total (t-) IκBα, β-actin, total (t-) and phosphorylated (p-) forms of p65 and p105 were detected by immunoblotting. For the immunoblotting, cell lysate (10 mg protein) was used. Representative results of t-IκBα, β-actin, p-p65, t-p65, p105 and t-p105 expressions (a), and the relative density of t- IκBα (b), p-p65 (c) and p-p105 (d) compared to the results at time point 0 (lower panel) are depicted. Values are expressed as the mean ± S.E. of three independent experiments. *P < 0.05. (e) Canine melanoma cells were exposed to 100 pM IL-1β for 15 min. At the end of the incubation, t-p65 (green) and lamin A/C (red; nuclei) were detected by immunocytochemistry.

In response to pro-inflammatory cytokines such as IL-1β, phosphorylation of the p65 subunit occurs, which has been shown to be important for the regulation of NF-κB transcriptional activity [36–41]. Phosphorylation of p105 protein in the cells stimulated with cytokines has also been shown to be involved in inflammation and cancer [37, 38, 40, 41]. Then, we examined the phosphorylation of the p65 subunit and p105 precursor in the cells treated with IL-1β. When the cells were treated with IL-1β, both p65 and p105 proteins were transiently phosphorylated, reaching peak levels at 15 min (Fig 3A–3C). To confirm whether NF-κB signaling was activated, we examined the degradation of IκBα in IL-1β-treated cells. As shown in Fig 3A and 3B, IL-1β induced the degradation of IκBα in a time dependent manner, suggesting that IL-1β activated NF-κB pathway.　 On the other hand, IL-1β had no effect on the activation of the other members of NF-κB family, RelB, c-Rel and p100 (S3 Fig), suggesting that p65 and p105 play a dominant role in IL-1β-induced COX-2 expression. It has been reported that p50 is generated by the 26S proteasome-mediated removal of C terminal consensus sequence of its precursor p105. In canine melanoma cells, the expression of p50 was observed without IL-1β treatment, and IL-1β had no effect on the expression of p50 (S2A Fig). In fact, in the cells transfected with siRNA for its precursor p105, the decrease of p50 expression was confirmed (S2B Fig). In addition, we examined IL-1β-induced nuclear translocation of p65 by immunocytochemical analysis. As shown in Fig 3E, the nuclear translocation of p65 was observed in the cells treated with IL-1β. Therefore, it is conceivable that IL-1β evoked the activation of NF-κB signaling in canine melanoma cells.

**Fig 4:**
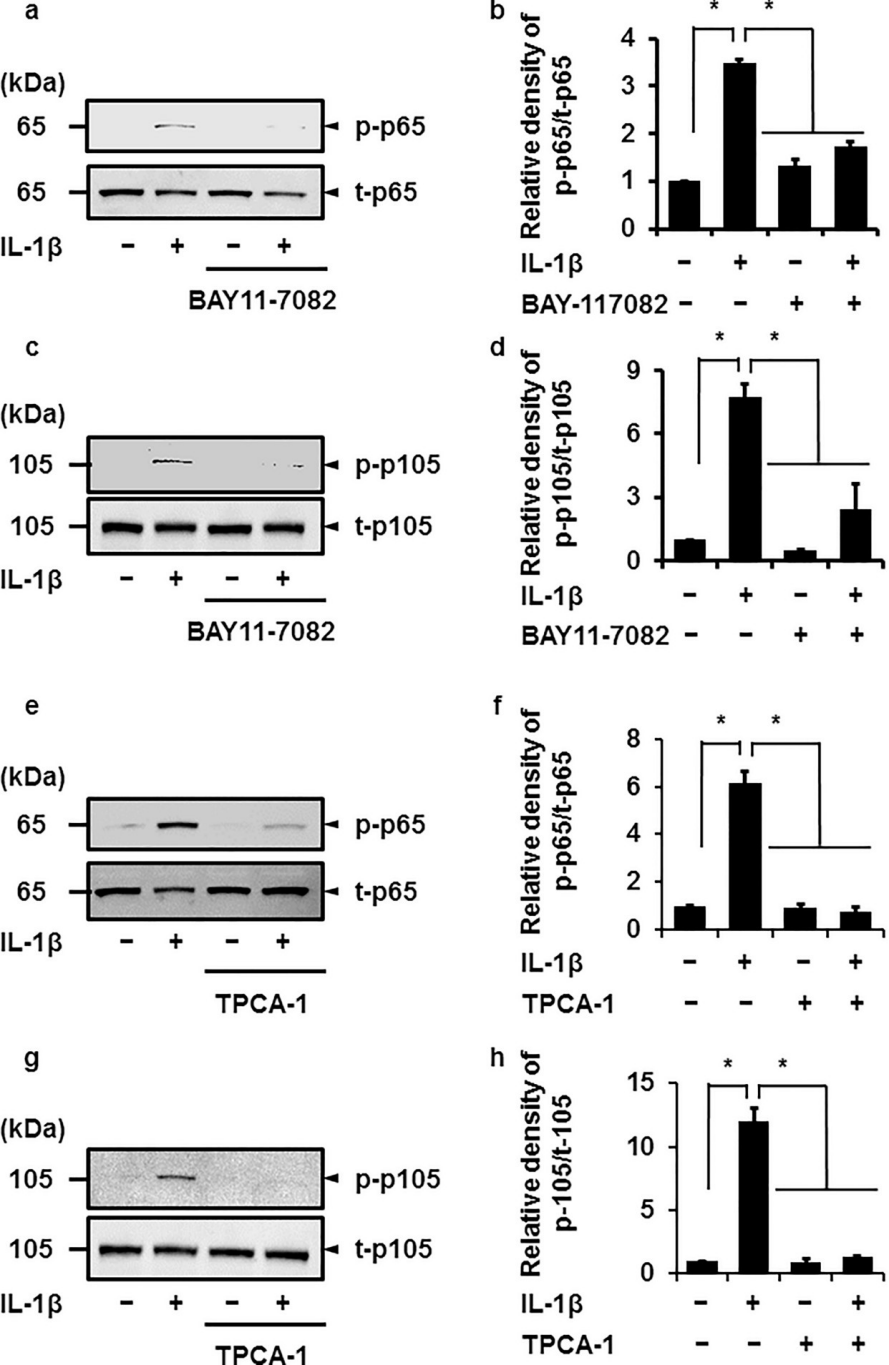
Inhibitory effect of NF-κB inhibitors on IL-1β-induced phosphorylation of p65 and p105. After pretreatment in the presence or absence of NF-κB inhibitors BAY 11–7082 (10 μM; a–d) and TPCA-1 (10 μM, e–h) for 1 h, canine melanoma cells were incubated with or without 100 pM IL-1β for 15 min, and then the phosphorylation levels of p65 and p105 were determined by immunoblotting. For the immunoblotting, cell lysate (10 μg protein) was used. Representative results (**a**, **c**, **e**, and **g**) and the relative density of p-p65 (**b** and **f**) or p-p105 (**d** and **h**) expression compared to the results without the inhibitor and IL-1β are illustrated. Values are expressed as the mean ± S.E. of three independent experiments. *P < 0.05.

When the cells pretreated with the NF-κB inhibitor BAY11-7082 or TPCA-1 for 1 h were stimulated with IL-1β for 15 min, IL-1β-mediated phosphorylation of both p65 or p105 proteins was clearly attenuated, as shown in Fig 4A–4H. These observations suggest that NF-κB, a heterodimeric complex consisting of p65 and p50, was involved in IL-1β-mediated functions in canine melanoma cells.

### Attenuation of IL-1β-mediated COX-2 mRNA expression in p65 or p105 knockdown cells

**Fig 5:**
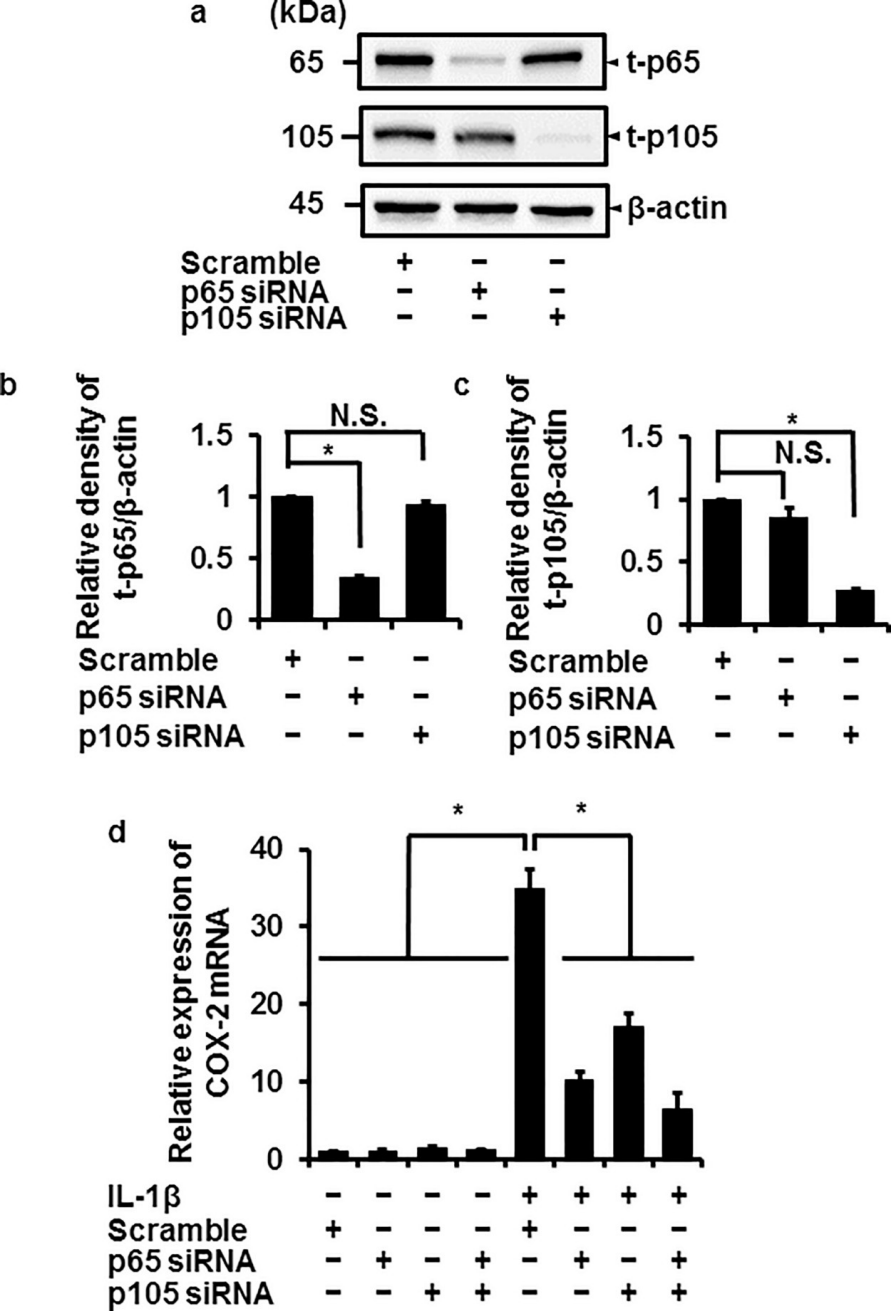
Inhibition of IL-1β-induced COX-2 mRNA expression in canine melanoma cells transfected with p65 and p105 siRNAs. Protein expressions of total p65 (t-p65), total p105 (t-p105), and β-actin (an internal standard) were detected by immunoblotting in canine melanoma cells transfected with p65, p105, or scramble siRNAs (control). For the immunoblotting, cell lysate (10 μg protein) was used. Representative results (**a**) and the relative density of t-p65 (**b**) and t-p105 (**c**) expression compared to the results in the control are illustrated. (**d**) The p65 and p105 siRNA transfection clearly inhibited the IL-1β-induced COX-2 mRNA expression. Values are expressed as the mean ± S.E. of three independent experiments. *P < 0.05. β-actin was used as an internal standard (a).

To elucidate the involvement of p65 and p105 in IL-1β-mediated expression of COX-2, we examined the effect of IL-1β on COX-2 mRNA expression in cells transfected with p65 and p105 siRNA. In cells transfected with p65 or p105 siRNAs, the expression of total p65 (t-p65) or p105 (t-p105) protein was clearly reduced compared with the control (cells transfected with scramble siRNA), respectively (Fig 5A–5C), similar to the reduction in IL-1β-induced COX-2 mRNA expression (Fig 5D). The reduced level of IL-1β-induced COX-2 mRNA expression in cells transfected with both p65 and p105 siRNAs showed no significant difference from that of p65 or p105 siRNA-transfected cells (Fig 5D). These observations suggest that p65 and p105 contribute to IL-1β-induced COX-2 mRNA expression in canine melanoma cells.

## Discussion

In this study, we demonstrated IL-1β-mediated COX-2 expression following prostaglandin E_2_ production in canine melanoma cells. The proinflammatory cytokine IL-1, including IL-1β, has been reported to be significantly elevated in melanoma [42]. IL-1β is secreted from immune cells, such as monocytes, macrophages, and dendritic cells [43]. Melanoma cells have also been demonstrated to spontaneously produce and release IL-1β, which leads to constitutive activation of the inflammasome [44]. Therefore, it is conceivable that IL-1β secreted from immune and melanoma cells in the tumor inflammatory microenvironment contributes to progression of cancer, including angiogenesis, invasion, and metastasis, via COX-2 expression and subsequent prostaglandin E_2_ production and release [45–47]. In fact, elevation of COX-2 expression is a common characteristic of various cancers, which mediates the progression and metastasis of tumors [48]. Regarding melanoma, the functional roles of COX-2 in invasion [49] and metastasis [50] have been proposed. COX-2 expression depends on both the stage and histopathologic type of melanoma [51, 52], and COX-2 expression has been suggested to be correlated with neoplastic recurrence and metastasis [53]. The COX-2-specific inhibitor celecoxib attenuated proliferation of melanoma cells, supporting that COX-2 is linked with melanoma progression [54].

In this study, we also demonstrated that NF-κB p65 and p105 are involved in IL-1β-mediated COX-2 expression in melanoma cells. NF-κB is a transcription factor that contributes to the regulation of a wide range of host genes involved in physiological and pathological functions. In cancer cells, NF-κB has been considered to play an important role for creating a favorable microenvironment to protect the cells against immune rejection and its promotion [55–57]. The NF-κB family is composed of five members, p65 (RelA), RelB, c-Rel, p50, and p52, which associate with each other to form homodimers or heterodimers with distinct functions [26]. Of these, the formation of the p50/p65 heterodimer is key to the activation of NF-κB [58, 59]. In the resting state of the cells, the p50/p65 heterodimer exists in the cytoplasm as an inactive complex form with the inhibitory protein IκBα. Furthermore, the NF-κB signaling pathway can be classified into canonical and noncanonical pathways [60]. In the canonical pathway, IκB kinase (IKK) is activated by exogenous signals such as IL-1β, which phosphorylates IκBα, inducing its ubiquitination and degradation by proteasomes. The heterodimer p50/p65 dissociated from IκBα translocates to the nucleus and transcriptionally regulates NF-κB target genes [60]. IL-1β-mediated COX-2 mRNA was reduced by selective inhibitors of IκBα and IKK, BAY11-7082 and TPCA-1, respectively, and was attenuated by the knockdown of p65 and p105 as the precursor of p50. These observations suggest that canonical activation of NF-κB signaling is important for IL-1β-mediated COX-2 expression in canine melanoma cells.

We observed that IL-1β transiently stimulated p65 phosphorylation in melanoma cells. The p65 protein contains an N-terminal Rel homology domain (RHD) and a C-terminal transactivation domain (TAD), and both domains and the linker region TAD to RHD have been reported to possess more than eleven phosphorylation sites [38, 40]. Phosphorylation of p65 occurs both in the cytoplasm and in the nucleus, and is thought as an important post-translational modification of NF-κB to efficiently induce transcription of target genes [38, 39]. Phosphorylation of individual amino acids has been related to effects of DNA binding, dimerization, association with transcriptional co-regulators, subcellular localization, stabilization, and transcriptional activity, which ultimately results in an increase or decrease in transcription depending on the amino acid modified [38–40, 61]. The transcriptional activity of NF-κB via phosphorylation sites of p65 depends on the stimulus. IL-1β has been demonstrated to regulate transcription of target genes via p65 phosphorylation [62–64]. Individual p65 phosphorylation induced by IL-1β have been considered to induce a conformational change and be regulated following association with transcriptional cofactors and ubiquitination [65]. Therefore, it is conceivable that phosphorylation of p65 is probably important for IL-1β-mediated COX-2 expression in melanoma cells, although further studies with specific phosphorylation sites are need.

Since IL-1β-mediated COX-2 mRNA expression was significantly reduced in p105-knockdown cells, it is conceivable that p105 is an important factor for translational activity in melanoma cells in response to IL-1β. Furthermore, p105 is a large precursor protein, which is partially processed by the proteasome to produce p50 subunit [24]. The p50 forms the p50/p65 heterodimer, which possesses transcriptional potential in the canonical pathway mediated by inflammatory signals [58, 59]. Therefore, it is most likely that p105 functions as the precursor of p50 in melanoma cells stimulated with IL-1β. Although p50/p65 is a dominant dimer, p50 can also form homodimers itself [66–68]. The p50/p50 homodimer exists in the nucleus, but cannot act as a transcriptional activator, since the subunit lacks a transactivation domain. Thus, the p50/p50 homodimer is thought to participate as the repressor in the NF-κB signaling [66–68]. Thus, such a function of p50 may be not neglectable.

We also observed that IL-1β transiently induced p105 phosphorylation. Therefore, IL-1β-induced phosphorylation appears to be involved in translational activity of NF-κB on COX-2 mRNA expression. Phosphorylation of p105 has previously been reported to be important for the proteasomal processing of p105 [69]. However, it has been considered that a majority of the p105 to p50 processing occurs co-translationally in a constitutive manner [70], and phosphorylation of p105 induces ubiquitination and results in the complete degradation of p105 without p50 generation [24, 71]. Currently, ubiquitination mediated by Kip1 ubiquitination-promoting complex 1 (KPC1), an E3 ubiquitin ligase, has been demonstrated to lead to proteasomal processing of p105 to p50, which is followed p105 phosphorylation by IKKβ [72]. However, the processing of p105 to p50 by KPC1 is involved in the downregulation of the NF-κB pathway, suppressing the progression of tumors including melanoma [72, 73]. On the other hand, p105 has also been demonstrated to have a role independent of the p50 precursor, which functions as a negative regulator in MAP kinase signaling [74, 75]. The p105 protein is bound to tumor progression focus 2 (Tpl-2), an apical kinase of the MAP kinase, for stabilizing Tpl-2 in an inactive form, which blocks the activation of MAP kinase signaling [74, 75]. The phosphorylation of p105 and subsequent proteasomal degradation of p105 results in the liberation of the active form of Tpl-2. Consequently, free Tpl-2 phosphorylates and activates MEK1/2, which induces the MAPK ERK1/2 activation [74, 75]. We previously reported that the NF-kB pathway contributes to ERK1/2 activation in canine dermal fibroblasts [76]. Therefore, such a regulation of p105 appears to be involved in IL-1β-mediated COX-2 expression in melanoma cells.

In conclusion, IL-1β mediated COX-2 expression and prostaglandin E_2_ release, in which NF-κB p65 and p105 functioned as transcriptional factors in canine melanoma cells. Thus, it is conceivable that the NF-κB pathway as well as IL-1β-mediated COX-2 expression is a therapeutic target for melanomas [18].

## Supporting information

S1 Fig Effects of IL-1β on cell viability. The viability of canine melanoma cells after incubation with (closed circles) and without (open circles) IL-1β (100 pM). Results are presented as mean ± SE from 3 independent experiments.
(PDF)

S2 Fig Expression of total p50 in canine melanoma cells (a) The cells were treated with IL-1β (100 pM) as the indicated time periods. IL-1β had no effect of the expression of total p50 (t-p50). (b) Protein expressions of total p50 (t-p50) were detected by immunoblotting in canine melanoma cells transfected with p65, p105, or scramble siRNAs (control). For the immunoblotting, cell lysate (10 μg protein) was used.
(PDF)

S3 Fig Effects of IL-1β on the phosphorylation of RelB, c-Rel and p100 in canine melanoma cells (a) The cells were treated with IL-1β (100 pM) as the indicated time periods. IL-1β had no effect of the phosphorylation of RelB, c-Rel and p100. For the immunoblotting, cell lysate (10 μg protein) was used.
(PDF)
