# Supplementary figures and images for: NF-κB p65 and p105 implicate in interleukin 1β-mediated COX-2 expression in melanoma cells
Source: PLoS One. 2018 Dec 18;13(12):e0208955. doi: 10.1371/journal.pone.0208955 (PMC6298655; doi:10.1371/journal.pone.0208955)

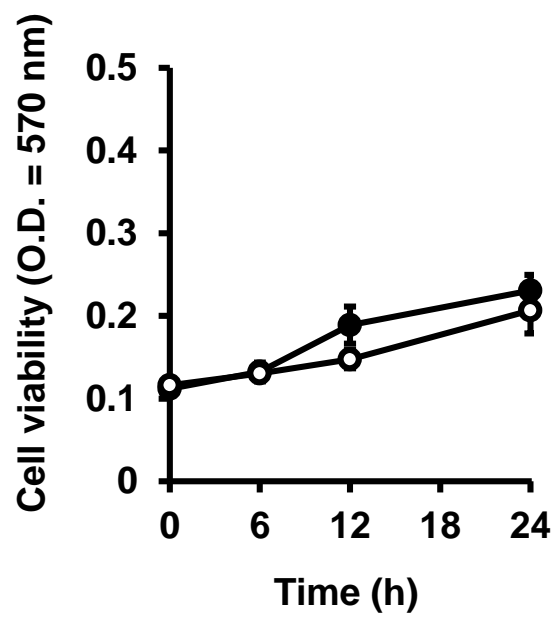

Supplement: S1 Fig — The viability of canine melanoma cells after incubation with (closed circles) and without (open circles) IL-1β (100 pM). Results are presented as mean ± SE from 3 independent experiments. (PDF) [file pone.0208955.s001.pdf]

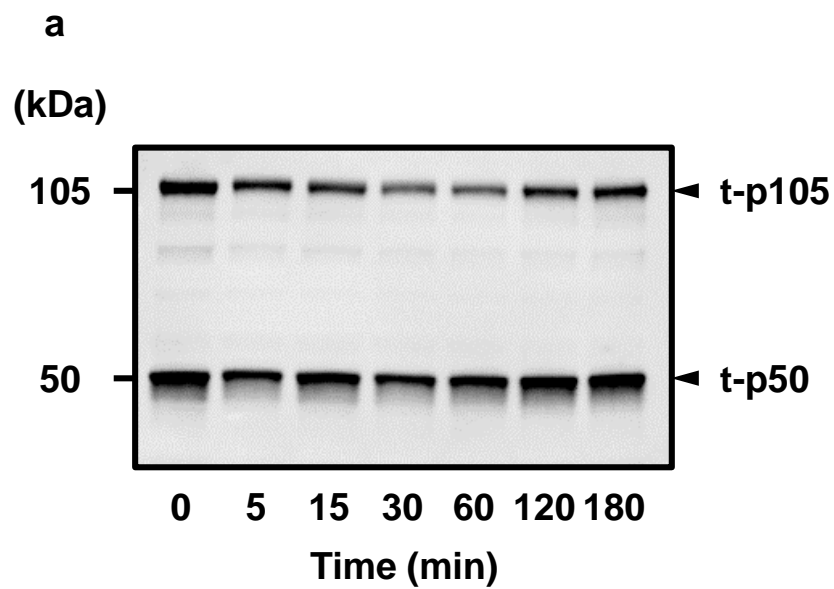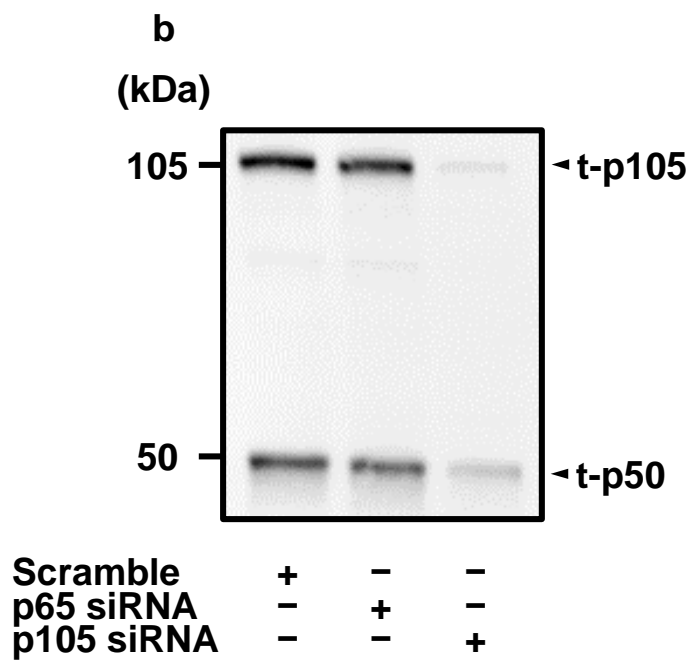

Supplement: S2 Fig — Expression of total p50 in canine melanoma cells (a) The cells were treated with IL-1β (100 pM) as the indicated time periods. IL-1β had no effect of the expression of total p50 (t-p50). (b) Protein expressions of total p50 (t-p50) were detected by immunoblotting in canine melanoma cells transfected with p65, p105, or scramble siRNAs (control). For the immunoblotting, cell lysate (10 μg protein) was used. (PDF) [file pone.0208955.s002.pdf]

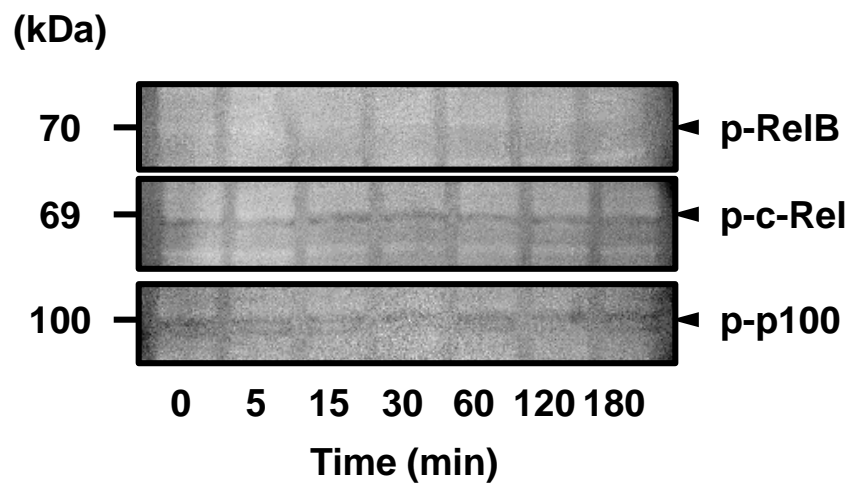

Supplement: S3 Fig — Effects of IL-1β on the phosphorylation of RelB, c-Rel and p100 in canine melanoma cells (a) The cells were treated with IL-1β (100 pM) as the indicated time periods. IL-1β had no effect of the phosphorylation of RelB, c-Rel and p100. For the immunoblotting, cell lysate (10 μg protein) was used. (PDF) [file pone.0208955.s003.pdf]
